# Toward a New Application of Real-Time Electrophysiology: Online Optimization of Cognitive Neurosciences Hypothesis Testing

**DOI:** 10.3390/brainsci4010049

**Published:** 2014-01-23

**Authors:** Gaëtan Sanchez, Jean Daunizeau, Emmanuel Maby, Olivier Bertrand, Aline Bompas, Jérémie Mattout

**Affiliations:** 1 Brain Dynamics and Cognition Team, Lyon Neuroscience Research Center, INSERM U1028-CNRS UMR5292, Lyon F-69000, France; E-Mails: manu.maby@inserm.fr (E.M.); olivier.bertrand@inserm.fr (O.B.); aline.bompas@inserm.fr (A.B.); jeremie.mattout@inserm.fr (J.M.); 2 University Lyon 1, Lyon F-69000, France; 3 Brain and Spine Institute, Paris F-75000, France; E-Mail: jean.daunizeau@gmail.com

**Keywords:** brain-computer interfaces, real-time electrophysiology, adaptive design optimization, hypothesis testing, Bayesian model comparison, Bayesian Decision Theory, generative models of brain functions, cognitive neuroscience

## Abstract

Brain-computer interfaces (BCIs) mostly rely on electrophysiological brain signals. Methodological and technical progress has largely solved the challenge of processing these signals online. The main issue that remains, however, is the identification of a reliable mapping between electrophysiological measures and relevant states of mind. This is why BCIs are highly dependent upon advances in cognitive neuroscience and neuroimaging research. Recently, psychological theories became more biologically plausible, leading to more realistic generative models of psychophysiological observations. Such complex interpretations of empirical data call for efficient and robust computational approaches that can deal with statistical model comparison, such as approximate Bayesian inference schemes. Importantly, the latter enable the optimization of a model selection error rate with respect to experimental control variables, yielding maximally powerful designs. In this paper, we use a Bayesian decision theoretic approach to cast model comparison in an online adaptive design optimization procedure. We show how to maximize design efficiency for individual healthy subjects or patients. Using simulated data, we demonstrate the face- and construct-validity of this approach and illustrate its extension to electrophysiology and multiple hypothesis testing based on recent psychophysiological models of perception. Finally, we discuss its implications for basic neuroscience and BCI itself.

## 1. Introduction

### 1.1. On Common Challenges in BCI (Brain-Computer Interfaces) and Cognitive Neurosciences

Brain-computer interfaces (BCIs) enable direct interactions between the brain and its bodily environment, as well as the outside world, while bypassing the usual sensory and motor pathways. In BCI, electroencephalography (EEG) is by far the most widely used technique, either with patients or healthy volunteers, simply because it offers a non-invasive, direct and temporally precise measure of neuronal activity at a reasonable cost [1]. BCI research is still mostly driven by clinical applications, and in this context, EEG has been used for a variety of applications. These range from replacing or restoring lost communication or motion abilities in patients suffering from severe neuromuscular disorders [2,3,4] and devising new therapies based upon neurofeedback training [5], to active paradigms in disorders of consciousness to better diagnose non-responsive patients [6] and possibly to communicate with those in a minimally conscious state [7]. Interestingly, common to most of these BCI objectives, but also to the ones in basic and clinical neurosciences, is the refinement of our understanding of the functional role of electrophysiological markers and their within- and between-subject variations. 

In this paper, we would like to further promote the idea that BCI and cognitive neuroscience researchers can help each other in pursuing this common goal. In short, the BCI paradigm puts the subject in a dynamic interaction with a controlled environment. From the perspective of cognitive neuroscience, this is a new opportunity to study normal and pathological brain functioning and to test mechanistic neurocognitive hypotheses [8]. In turn, BCI can benefit from progress in neurocognitive models for decoding mental states from online and single-trial electrophysiological measures [9]. Taking BCI outside the laboratory for daily life applications with patients or healthy people raises tremendous challenges, one of which is the need to decode brain signals in real time. This means one has to be capable of making efficient and robust inference online based on very limited, complex and noisy observations. Large efforts have recently been put into developing and improving signal processing, feature selection and classification methods [10,11,12], as well as acquisition hardware techniques [13] and dedicated software environments [14,15]. However, the main BCI bottleneck consists in the identification of a reliable mapping from neurophysiological markers to relevant mental states. This unresolved issue advocates for tight collaborations between BCI developers, electrophysiologists and cognitive neuroscientists. 

Thankfully, a recent trend (and one that is increasingly catching on) has been to increase the permeability of the border between the BCI and cognitive neuroscience communities. New applications have emerged that rely on both disciplines and, thus, bring short-term benefit to both. One example is the so-called brain-state-dependent stimulation approach (BSDS) [16], the principle of which is to use BCI as a research tool for cognitive neuroscience, namely to study causal relationships between brain state fluctuations and cognition. In the BSDS, the functional role of a brain state is studied by delivering stimuli in real time to subjects, depending on their brain’s actual physiological state. Other examples illustrate the reverse direction of this putative multidisciplinary cross-fertilization, showing how advances in cognitive neuroscience may improve BCI performance. An example is connectivity model-based approaches to neurofeedback, as demonstrated recently using fMRI (functional Magnetic Resonance Imaging) [17]. It is to be noted that such emerging applications tend to extend the usefulness of BCI and real-time data processing to non-invasive techniques other than EEG, such as fMRI and MEG (Magnetoencephalography), which have similar overall principles, but might be even more effective for answering some of the cognitive neuroscience questions. 

In this paper, we extend and formalize the BSDS approach by showing that our ability to process neuroimaging data online can be used to optimize the experimental design at the subject level, with the aim of discriminating between neurocognitive hypotheses. In experimental psychology and neuroimaging, this is a central issue, and examples range from stair-case methods to estimating some individual sensory detection or discrimination threshold [18], to design efficiency measures to optimize the acquisition parameter or the stimulus onset asynchrony (SOA) in fMRI studies [19]. The former operates in real time in the sense that the next stimulation depends on the previous behavioral response and is computed in order to optimize model fitting. The latter operates offline, prior to the experiment, and its aim is to optimize model comparison.

### 1.2. Adaptive Design Optimization

**Figure 1:**
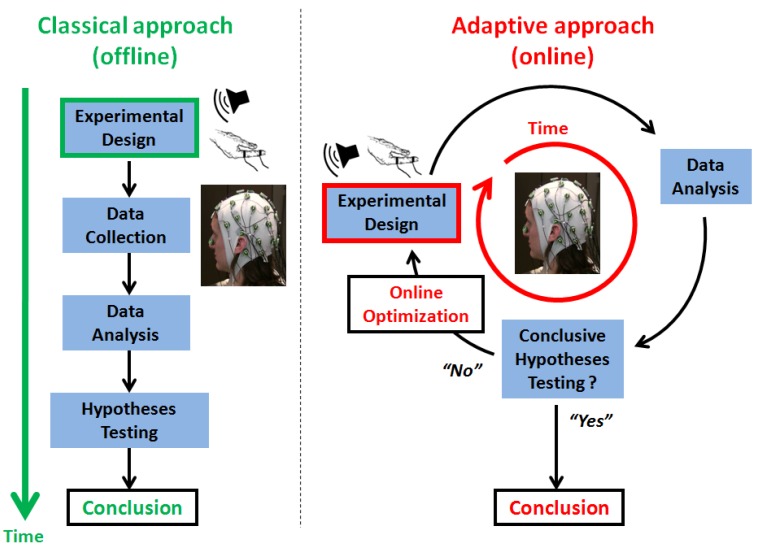
A schematic illustration of the adaptive *versus* classical experimental design approaches. The classical approach (*left*) is characterized by a sequential ordering of the main experimental steps: experimental design specification occurs prior to data acquisition, which is followed by data analysis and hypothesis testing. In contrast, the adaptive approach (*right*) operates in real time and proceeds with design optimization, data acquisition and analysis at each experimental stage or trial. The online approach enables hypothesis testing to be optimized at the individual level by adapting the experimental design on the basis of past observations. This is the general principle of adaptive design optimization (ADO), which can be extended to advanced computational models of electrophysiological responses thanks to brain-computer interface (BCI) technology, with the aim of optimizing experimental conclusions and the time-to-conclusion in cognitive and clinical neuroscience.

We introduce a generic approach in which real-time data acquisition and processing is aimed at discriminating between candidate mappings between physiological markers and mental states. This approach is essentially an adaptive design optimization (ADO) procedure [20]. The origins of ADO stem back to sequential hypothesis testing methods [21], whose modern forms have proven useful in human, social and educational sciences, where typical experiments involve a series of questions to assess the level of expertise of a particular subject [22]. The general principle is fairly straightforward. Figure 1 illustrates its application in the context of human electrophysiology and neuroimaging. In contrast with standard (non-adaptive) experiments, in ADO, the total number of trials is not set in advance, nor is the nature of the stimulation at each trial or stage of the experiment. Moreover, one does not wait until the end of the data acquisition process to proceed with data analysis and statistical inference. Instead, for each trial, the appropriate data features are extracted in order to up-date our (the experimenter’s) information about the model parameters and to assess the model plausibility itself. Based on these estimates, a decision is made regarding some relevant design parameters for the next trials. The decision criterion should reflect the scientific objective of the experiment, e.g., a target statistical power for parameter estimation. This implies that some threshold can be met that would terminate the current experiment. In other words, ADO behaves like classical approaches, except that it operates online, at each trial. In turn, incoming trials are considered as future experiments, whose design can be informed by past observations or simply become unnecessary. At the level of a single subject, ADO can be used to improve on three problems: (i) model parameter estimation; (ii) hypothesis testing *per se*; (iii) the duration of the experiment. In the fields of experimental psychology and electrophysiology, recent forms of ADO have been applied to estimating psychometric functions [23], optimizing the comparison of computational models of memory retrieval [24] and optimizing the duration of the experiment when comparing alternative neuronal models [25]. However, optimizing parameter estimation and hypothesis testing do not call for the same criteria and might not be possible simultaneously. In this paper, we focus on ADO for optimizing model comparison, which appears to be of primary interest in cognitive neuroscience. This is because, over the past decade, dynamic and non-linear computational models of neuroimaging and behavioral data have been flourishing [26]. In particular, established control theoretic approaches now rely upon biologically and psychologically plausible models of fMRI, electrophysiological or behavioral data (see, e.g., dynamical causal models (DCMs); [27,28,29]). Such generative models aim to explain the causal relationship between experimental (e.g., cognitive) manipulations and the observed neurophysiological or behavioral responses [30]. In particular, such tools have now been used to compare alternative models of learning and decision making in humans [28]. Importantly, these models are embedded in a Bayesian statistical framework, which allows one to deal with complex (e.g., probabilistic) models by introducing prior knowledge about unknown model parameters. Note that statistical inference can be made quick and efficient through the use of generic approximation schemes (*cf*. variational Bayes approaches; [31]). To extend ADO to dynamical neurocognitive models of electrophysiology data, we bring together such variational Bayesian approaches (which can be used in real time) and recent advances in design optimization for Bayesian model comparison (which can deal with complex models; [32]).

This paper is organized as follows. In the Theory and Methods section, we first describe the class of dynamical models that we compare. To make this paper self-contained, but still easy to read, we provide an appendix with a comprehensive summary of the variational Bayesian inference approach (see Appendix A1) and the design efficiency measure (see Appendix A2) that we rely on, in this new instantiation of ADO. We also emphasize how this compares with the recent pioneering approach for ADO in experimental psychology [20,24]. In the second part of the methods section, we introduce our validation strategy, which consists first of a demonstration of the face and construct validity of our approach by considering the same behavioral example as in [20]. Continuing to use synthetic data, we then demonstrate the extension of our approach to comparing variants of recent dynamical models of perceptual learning. In particular, by simulating several subject datasets, we illustrate how ADO compares with classical designs and how it optimizes hypotheses at the individual level. The next section presents the results of this validation. In the last section, we discuss these results, the perspectives they offer, as well as the challenges we now face to put ADO into practice.

## 2. Theory and Methods

### 2.1. Dynamic Causal Models (DCMs)

In this section, we briefly introduce the very general type of complex generative models for which the proposed ADO procedure is most appropriate. In their general form, such models are defined by a pair of assumptions {*f*, *g*}. The first component, *f*, is the evolution function, which prescribes the evolution or motion of hidden (unobservable) neuronal or psychological states *x*, such that:

*x* = *f*(*x*, *θ*, *u*) (1)

The second component, *g*, is the observation function and prescribes the mapping from hidden states to observed neurophysiological, metabolic or behavioral responses, such that:

*y* = *g*(*x*, *φ*, *u*) + *ε* (2)

*θ* and *φ* are the model parameters. They represent fixed, but unknown, values that parameterize the evolution and observation functions, respectively. These values might differ from one subject to another or, for the same subject, from one experimental condition to the next. *ε* indicates random fluctuations or noise that corrupt the observed data. Finally, *u* corresponds to experimental control variables, that is, exogenous inputs to the system that might encode changes in experimental condition (e.g., visual stimulation-type, like face *vs.* house) or the context under which the responses are observed (e.g., sleep *vs.* awake). Instantiations of such models have been proposed to explain the generation and the effect of experimental modulations in fMRI data [27] and various electrophysiological features in EEG, MEG or intracranial (*i.e.*, local field potentials (LFP)) data, such as evoked [33], induced [34] or steady-state responses [35].

More recently, a related dynamical-system based approach has been derived to model psychological states, their evolution over time and their mapping onto observable behavioral measures (e.g., choices, reaction times) [28] or physiological observations [36]. However, referred to as “observing the observer”, this approach differs from the above classical DCMs, because it involves the embedding of a subject’s (the observer) dynamic causal model of the environment (*M_s_* = {*f_s_*, *g_s_*}) into an experimenter’s (another observer observing the subject) dynamic causal model of the subject (*M_e_* = {*f_e_*, *g_e_*}). Further, assuming that the subject implements an optimal online Bayes inference (see Appendix A1) to invert the duplet {*f_s_*, *g_s_*} and infer the hidden states of the environment, the evolution (perception) function, *f_e_*, incorporates this inference and learning process, while the observation (response) function, *g_e_*, defines the mapping between the hidden subject’s internal states (the inferred or posterior estimates of the environment hidden states) onto behavioral or physiological responses. Bayesian inference applies to the experimenter’s model in order to compare pairs of models {*M_s_*, *M_e_*}and infer those model parameters (see Appendix A1). This is why this approach is also referred to as a meta-Bayesian approach [28]. Importantly, in this context, we explicitly model the link between the precise sequence of presented sensory inputs and the evolving subject’s beliefs about the state of the world.

### 2.2. Online Optimization of Model Comparison

Most of the generative models that are used in cognitive neuroscience fall into the class of nonlinear Gaussian models. Our approach combines two recent methodological advances and brings them online for ADO. First, we use a Bayesian framework to invert and compare such generative models [28] (see Appendix A1). Second, we use a previously proposed proxy to the model selection error rate [32] as a metric to be optimized online through the appropriate selection of experimental control variables (see Appendix A2). Under the Laplace approximation [37], this metric (the Chernoff bound) takes a computationally efficient analytic form, which is referred to as the Laplace–Chernoff bound. In [32], the authors disclosed the relationship between the Laplace–Chernoff bound and classical design efficiency criteria. They also empirically validated its usefulness offline, in a network identification fMRI study, showing that deciding whether there is a feedback connection between two brain regions requires shorter epoch durations, relative to asking whether there is experimentally-induced change in a connection that is known to be present.

For the online use of the same criterion in order to optimize the experimental design for model comparison, at the individual level, we simply proceed as illustrated in Figure 1 in the adaptive scenario. At each trial or experimental stage, it consists of:

(i) Running the variational Bayes (VB) inference for each model, *M*, given past observations and experimental design variables;
(ii) Updating the prior over models with the obtained posteriors;
(iii) Computing the design efficiency or Laplace-Chernoff bound for each possible value of the experimental design variable, *u*;
(iv) Selecting the optimal design for the next trial or stage.

Finally, the online experiment will be interrupted as soon as some stopping criterion will have been met. Typically, the experiment will be conclusive as soon as one model is identified as the best model, for instance, when its posterior probability will be greater than 0.95. If this is not the case, when an *a priori* fixed number of trials would have been reached, the experiment will be considered as inconclusive in selecting a single best model for the given subject.

### 2.3. Validation

We now turn to the validation of the proposed approach. We describe two studies based on synthetic data. The first one demonstrates the face and construct validity of the approach by reproducing the simulation example in [20]. The second study illustrates how our approach extends to a realistic online scenario, whose aim is to compare more than two nonlinear models of perceptual learning based on electrophysiological responses only.

#### 2.3.1. First Study: Synthetic Behavioral Data

In order to illustrate our approach for ADO and to provide a first demonstration of its face and construct validity, we reproduce results from Cavagnaro and colleagues [20,38]. These authors showed how an optimal design might look in practice, considering the example of a typical behavioral experiment designed to discriminate psychological models of retention (*i.e.*, forgetting). The experiment consists of a “study phase”, in which participants are given a list of words to memorize, followed by a time interval (lag time), followed by a “test phase”, in which retention is assessed by testing how many words the participant can correctly recall from the study list. The percentage of words recalled correctly typically decreases with the time interval. A model of retention is the function that can fit this relationship between retention and lag time. These authors considered two retention models: power and exponential forgetting [38].

Model power (POW):

*p* = *a*(*t* + 1) ^−*b*^ (3)

Model exponential (EXP):

*p* = *ae*^−^*^bt^* (4)

In each equation, the symbol, *p*, denotes the predicted probability of correct recall as a function of lag time, *t*, between the study and test phase, with model parameters *a* and *b*.

As in [38], we simulated data under the (true) model POW, considering plausible values for model parameters. Note that the retention interval or lag time is the design variable whose value is being experimentally manipulated. For a given lag time, *t*, each model predicts the number of correctly recalled items:

*y* = *n.a.* (*t* + 1) ^−*b*^ (5)

where *n* = 30 is the number of presented items at each trial.

The observable data, *y*, in this memory retention model formally follows a binomial distribution and (conjugate) Beta priors on parameters (*a*,*b*) are usually used. In our case, we used a normal approximation to the priors on parameters (*a*,*b*). As *n* increases, according to the central limit theorem, the binomial distribution tends to a normal density with matched moments, and a normal approximation to the likelihood function is appropriate. We simulated the responses from 30 participants, by drawing 30 pairs of parameter values *a* and *b*, considering *a* ~ Ɲ(0.8,0.5) and *b* ~ Ɲ(0.4,0.5).

For each simulated participant, ADO was initialized with the same priors over model parameters: *a* ~ Ɲ(0.75,2), *b* ~ Ɲ(0.85,2) for POW and *a* ~ Ɲ(0.9,2), *b* ~ Ɲ(0.15,2) for EXP; and the same prior for each model: *p*(POW) = *p*(EXP) = 1/2. Similar to what Cavagnaro and colleagues did, we compared ADO against two classical (non-adaptive) experimental designs. The first one, called “Random Design”, is a complete random fashion design, where the lag time at each trial was chosen randomly between 0 and 100 s. The second one, called “Fixed 10 pt Design”, presents, in a random order, each lag time from a fixed set of lag times concentrated near zero and spaced roughly geometrically: 0, 1, 2, 4, 7, 12, 21, 35, 59 and 99 s. The latter design is closer to the set of lag times used in real retention experiments [39]. We considered 10 trial-long experiments and computed the true (POW) model posterior after each trial, for each design. Only ADO is adaptive in the sense that, at each trial, the most efficient lag time is selected based on the updated posteriors over parameters and models, and the ensuing Laplace-Chernoff bound for each possible lag times. The results are presented in Section 3.1.

#### 2.3.2. Second Study: Synthetic Electrophysiological Data

To demonstrate how our new instantiation of ADO extends to nonlinear dynamic causal models, which are of increasing interest in cognitive neuroscience, we now turn to a second series of original simulations. We therefore consider recent models of human perceptual learning in a changing environment [40,41,42,43] and combine them with recent works on how these models might predict single-trial EEG evoked responses [36,44]. These models can be thought of as a specific instantiation of the Bayesian brain and predictive coding hypotheses [45]. The former hypothesis postulates that the brain uses Bayesian inference for perception and perceptual learning. In other words, these processes rely upon an internal generative model, *i.e.*, probabilistic assumptions of how external states cause changes in sensory data (the sensory signal likelihood) and prior beliefs about these causes [46]. In addition, the predictive coding hypothesis [47] suggests that electrophysiological activity that propagates through neural networks encodes prediction (top-down) and prediction error (bottom-up) messages, whose role is to explain away sensory surprise by updating beliefs about hierarchically deployed hidden causes. Evoked electrophysiological responses that are reminiscent of such mechanisms were first established using so-called “oddball” experimental paradigms, where one category of rare stimuli (deviants) is intermixed with a second category of frequent stimuli (standards). The ensuing “mismatch negativity” (MMN) EEG evoked potential is then interpreted in terms of the response of the system to a violation of its prior expectations [48]. These responses have been observed in various sensory modalities, but are mostly documented in the auditory [49] and somatosensory domains [44].

Below, we expose the perceptual (evolution) and response (observation) models we considered for simulating MMN-like responses.

#### 2.3.2.1. Perceptual Learning Model

**Figure 2:**
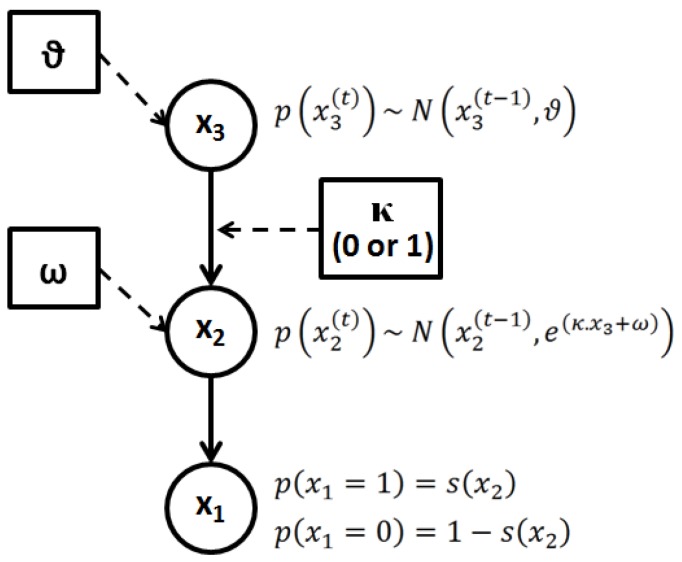
Graphical illustration of the hierarchical perceptual (generative) model with States *x*_1_, *x*_2_ and *x*_3_. The probability at each level is determined by the variables and parameters at the level above. Each level relates to the level below by controlling the variance of its transition probability. The highest level in this hierarchy is a constant parameter, *ϑ*. At the first level, *x*_1_ determines the probability of the input stimulus: standard (0) or deviant (1). The model parameters, *ω* and *ϑ*, control the agent’s belief update about State *x*. Note that setting *κ* = 0 effectively truncates the hierarchy to the first two levels. In the diagram, squares represent fixed parameters, while circles represent state variables that evolve in time.

We considered a simplified version of the perceptual learning model proposed in [43] to model perception in a volatile environment (see also [50]). This perceptual model (Figure 2) comprises a hierarchy of 3 hidden states (denoted by *x*), with States 2 and 3 evolving in time as Gaussian random walks. The probability of a stimulation category appearing in a given trial (*t*) (represented by State 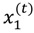, with *x*_1_ = 1 for deviant and *x*_1_ = 0 for standard stimuli) is governed by a state, *x*_2_, at the next level of the hierarchy. The brain perceptual model assumes that the probability distribution of *x*_1_ is conditional on *x*_2_, as follows:

*p*(*x*_1_│*x*_2_) = *s*(*x*_2_)^*x*_1_^ (1 − *s*(*x*_2_)) ^1−*x*_1_^ = *Bernoulli* (*x*_1_;*s*(*x*_2_)) (6)

where *s*(∙) is a sigmoid (softmax) function:

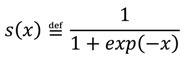
(7)

Equations (6) and (7) imply that the states *x*_1_ = 0 and *x*_1_ = 1 are equally probable when *x*_2_ = 0.

The probability of *x*_2_ itself changes over time (trials) as a Gaussian random walk, so that the value, 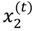, is normally distributed with mean 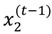 and variance 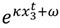:

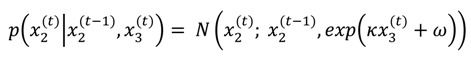
(8)

Setting the parameter *κ* to 0 effectively means assuming that the volatility of *x*_2_ is fixed over time. In all other cases, the magnitude of changes in *x*_2_ over time (trials) is controlled by *x*_3_ (the third level of the hierarchy) and *ω*, which can be regarded as a base (log-) volatility. The state, 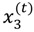, on a given trial is normally distributed around 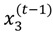, with a variance determined by the constant parameter, *ϑ*. The latter effectively controls the variability of the log-volatility over time.


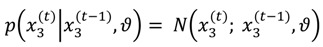
(9)

#### 2.3.2.2. Electrophysiological Response Model

One can quantify the novelty of sensory input using Bayesian surprise. In what follows, we assume that EEG response magnitudes encode the Bayesian surprise induced by the observation of sensory stimuli at each trial. This is in line with recent empirical studies of the MMN in oddball paradigms [36,44].

Recall that, at any given trial, the Bayesian surprise is simply the Kullback-Leibler divergence between the prior and posterior distribution [51]. It indexes the amount of information provided by sensory signals at each level of the hierarchy. We simulated trial-by-trial EEG response magnitudes by adding random noise to the (weighted) Bayesian surprise (BS) at the second level of the perceptual learning model:

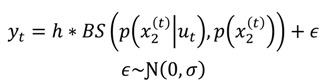
(10)

Note that under the Laplace approximation, BS has a straightforward analytic form (see [52]). In the current simulations, we fixed the weight parameter, *h*, to −10 and the noise precision or inverse variance to 100.

We considered the problem of comparing five different perceptual models given simulated EEG data (see Table 1). M1 is a “null” model with no learning capacities. The four other models form a 2 × 2 factorial model space. Contrary to M4 and M5, M2 and M3 have no third level (*κ* = 0). They are unable to track the volatility of the environment. Orthogonal to this dimension is the base learning rate at the second level, which is controlled by the parameter, *ω*. In brief, M2 and M4 predict slower learning than M3 and M5.

**Table 1:** Five alternative models used and compared in simulations.

Models	*ω* Values	*κ* Values (If *κ* = 0, No Third Level)	*ϑ* Values	Ability to Track Events Probabilities	Ability to Track Environmental Volatility
M1	−Inf	0	-	No	No
M2	−5	0	-	Low learning	No
M3	−4	0	-	High learning	No
M4	−5	1	0.2	Low learning	Yes
M5	−4	1	0.2	High learning	Yes

We simulated 75 experiments in total, corresponding to 15 different synthetic subjects simulated under each model type as the true model. Each experiment consists of 350 trials. ADO was compared with the two following classical designs. The “stable” classical design has a fixed probability of the occurrence of a deviant (*p*(*u* = 1) = 0.2). The “volatile” classical design starts with 100 trials with a stationary sensory signal distribution (*p*(*u* = 1) = 0.2), followed by 150 trials with a volatile sensory signal distribution (which alternates 50 trials with *p*(*u* = 1) = 0.1, 50 trials with *p*(*u* = 1) = 0.3 and 50 trials with *p*(*u* = 1) = 0.1), followed by a stable period similar to the initial one. Results are presented in Section 3.2.

### 2.4. Software Note

Simulations in this work were performed using the VBA toolbox [53], which is under open-source GNU General Public License (v2) and freely downloadable from the toolbox’s internet wiki pages [54].

## 3. Results

### 3.1. First Study: Behavioral Synthetic Data

**Figure 3:**
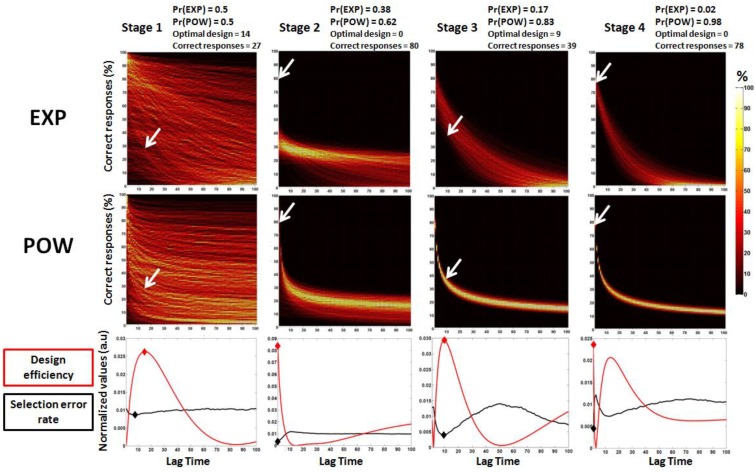
Predictions of the power (POW) and exponential (EXP) models in the first four stages of one simulated experiment and the landscape of selection error rate across lag time. The predictions are based on the prior parameter estimates at each stage. The text above and inside the graphs provides information about the prior probabilities of each model, the optimal designs for discriminating the models and the observed outcomes (correct responses) at each stage of the simulated experiment. Arrows denote the percentage of correct responses at the optimal lag time. For the heat maps of models predictions (*top* and *middle* panels), yellow colors indicate regions of higher probability. (*Bottom*) The bottom panel, represents the error selection rate for each possible lag time (normalized values of arbitrary units, *black line*), as well as the estimated efficiency (*red line*), which is our main criterion. At each stage, we choose the maximum of our criterion (*red diamond*), which mostly coincides (because of the approximation) with the minimum of the error selection rate (*black diamond*).

In brief, ADO chooses, at each stage, the lag time that maximizes the difference between model predictions and then updates model probabilities based on the model evidences. For example, in Stage 3 of the simulated experiment depicted in Figure 3, the optimal time lag was around 9 s. At this time lag, EXP predicts a higher percentage of correct responses than POW (*cf*. heat maps). When 39% of correct responses are observed (an outcome that is much more likely under POW than under EXP, *cf*. white arrows in Figure 3), POW’s posterior probability is increased from 0.83 to 0.98 in Stage 4. Instead, EXP’s posterior probability decreases from 0.17 to 0.02. As the experiment unfolds, the models’ predictions converge towards the observed outcomes and the posterior probability of the true model (POW) approaches 1.

**Figure 4:**
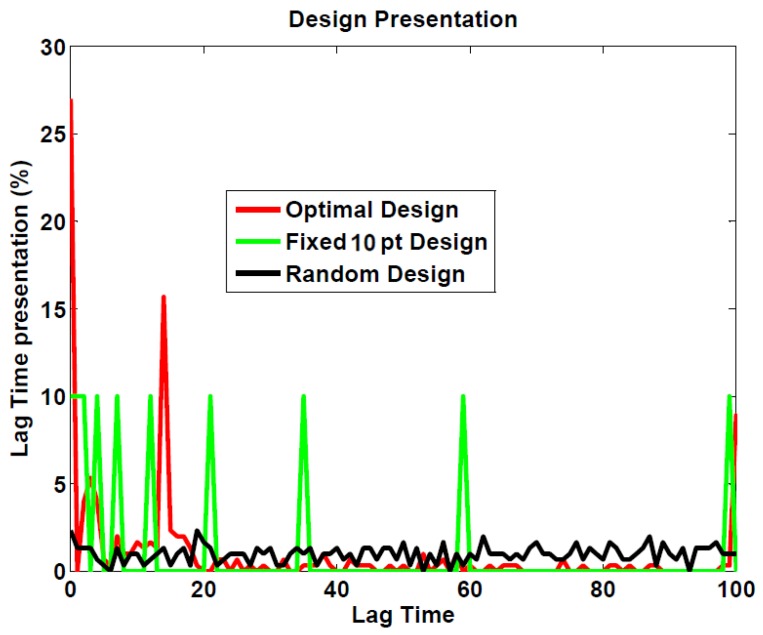
Lag time distribution for each experimental design (over the 30 simulations).

In line with Cavagnaro *et al.*, we compared ADO with two random classical designs. Figure 4 shows the distribution of lag time presentations for ADO and for both random designs (simulations with group size = 30). One can see that ADO selects lag times that lie at the extremes of the permitted range, or between 10 and 20 s. Given those distributions, the expected mean lag time per stage for each design is: ADO: 19.2; fixed 10 pt design: 24; and random design: 49.3.

**Figure 5:**
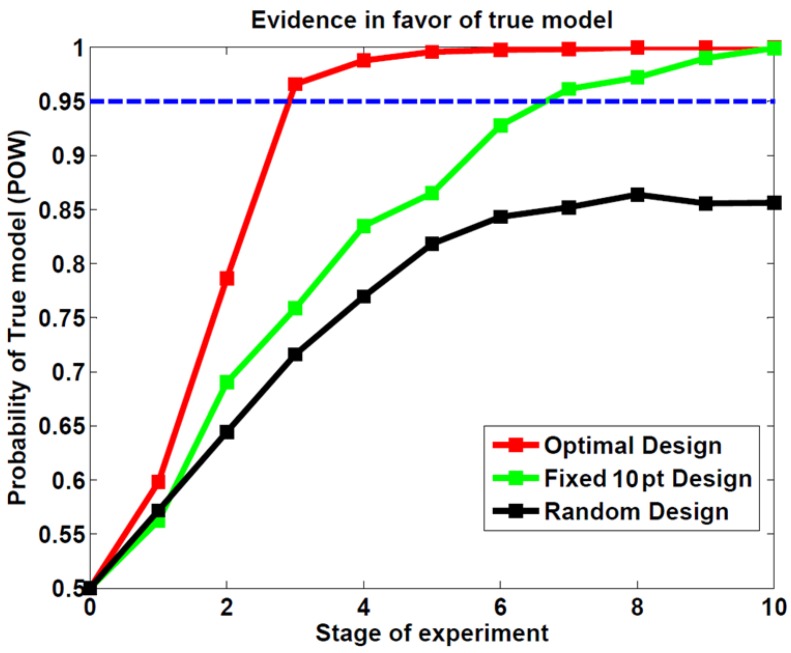
Posterior probabilities of the true (POW) model at each stage (average over 30 simulations).

Finally, Figure 5 shows the group mean of posterior probability for the true model (POW) as a function of stage depending on the design. On average (over the 30 subjects), ADO reaches a posterior model probability of 95% after about three stages. On average, the random design was still inconclusive after 10 stages (the posterior model probability is still below 95%). The fixed 10 pt design reaches a posterior model probability of 95% after seven stages, which corresponds to experiments twice as long as with ADO.

In brief, our analysis reproduces the results of Cavagnaro *et al* [38]. Note that here, the optimal design was easily found using the direct calculation of the minimum model selection error rate (the black line in Figure 3). Although this may render our results rather anecdotic, our intention was simply to validate the approach on a simple case. In what follows, we generalize our results to a much more complex (and realistic) design optimization problem in which the direct calculation of the minimum model selection error rate is impossible.

### 3.2. Second Study: Electrophysiological Synthetic Data

**Figure 6:**
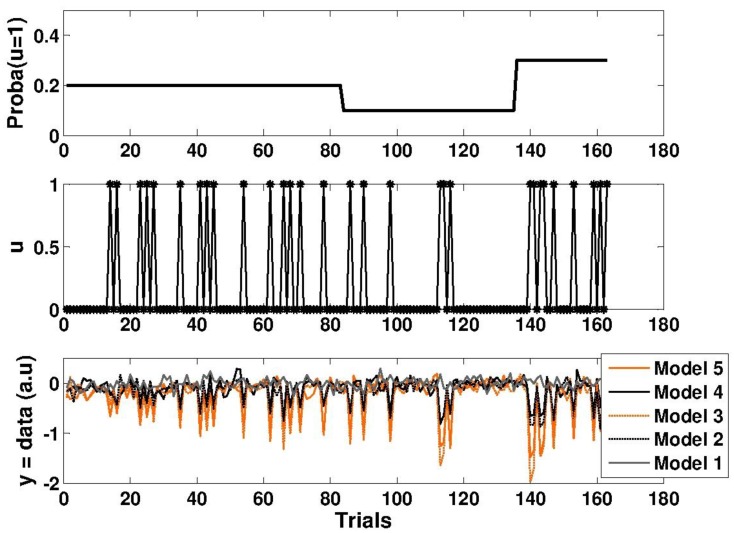
Simulated data for five Bayesian learning models (defined in Table 1). (*Top*) The dynamics of true deviant probability. (*Middle*) The sequence of sensory stimuli u (0 or 1). (*Bottom*) The dynamics of (noisy) Bayesian surprise (simulated electroencephalography (EEG) response magnitudes over trials).

Here, we assess ADO’s ability to discriminate between complex (computational) models. Figure 6 presents an example of a simulation for each of the five models described in the results section (see Table 1). Under the “null” model (M1), fluctuations in the data are explained by measurement noise. One can see early differences in simulated data under models with high (M3 and M5) or low (M2 and M4) learning rates (the *ω* parameter). However, differences between three- (M4 and M5) and two- (M2 and M3) level models only appear when the sensory signal distribution becomes volatile.

**Figure 7:**
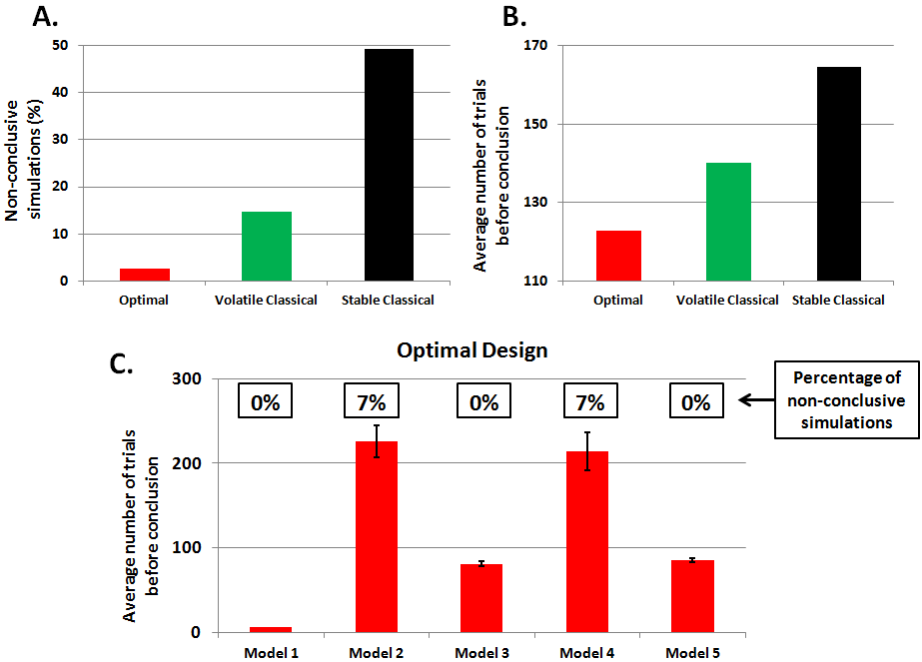
Adaptive design optimization (ADO) with learning models: simulation results. Note that a simulated experiment is deemed “conclusive” whenever the true model posterior probability is equal to or greater than 95%. (**A**) The number of non-conclusive experiments for each design; (**B**) the average number of trials needed to reach the 95% threshold in conclusive simulations; (**C**) the average number of trials before the conclusion and the percentage of non-conclusive simulations (note that in our case, 7% means one non-conclusive experiment over 15).

As in the previous Section 3.1, we assessed the designs’ ability to discriminate between the candidate models. Figure 7 summarizes the results over 75 simulations (with equal proportions of datasets simulated under each model). We considered that an experiment was conclusive when the posterior probability in favor of the true model reached or exceeded the threshold of 0.95 (after 350 trials). The simulation is labeled “non-conclusive” otherwise. We observed that almost half of the simulations (49.3%) were labeled non-conclusive when we used the stable classical design. The volatile classical design was much more efficient (14.7% of non-conclusive experiments), but less than ADO (2.7% of non-conclusive experiments). When focusing on conclusive experiments, we observed differences in the number of trials needed to reach the 95% posterior model probability threshold. In brief, ADO yields faster experiments than both the volatile and stable classical designs (see Figure 7).

**Figure 8:**
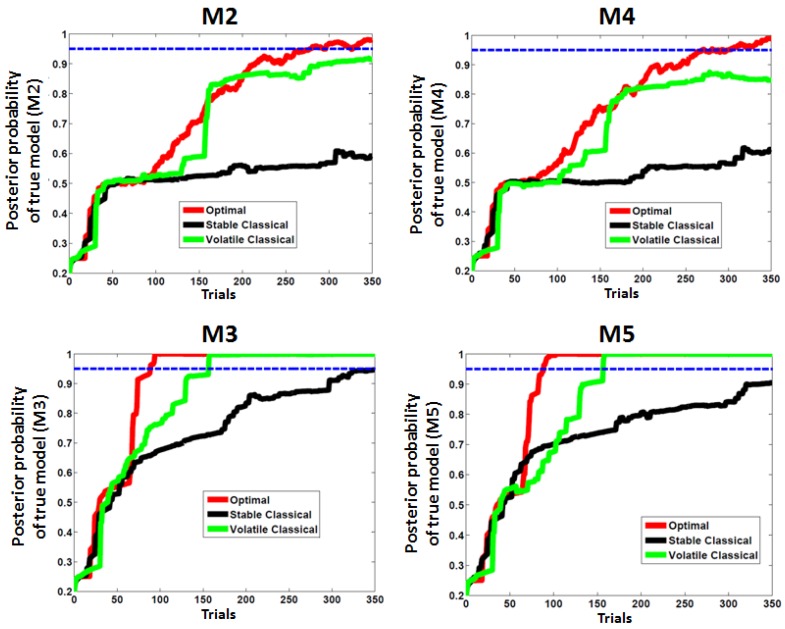
Posterior model probabilities at each trial in our simulated experiment: the average over 15 simulations for each model.

Figure 8 shows the average dynamics of model posterior probabilities, as a function of the true model. Note that M1 was discarded by all designs after about 10 trials (not shown). One can see that only ADO can select models with low learning rates (M2 and M4), given the 95% model posterior probability requirement. Note that models with higher learning rate (M3 and M5) take fewer trials to be selected, although the stable classical design only reaches the 95% threshold at the very end of the experiments for M3.

In conclusion, ADO performs better than classical designs, yielding fast and efficient experiments for all the models considered. The last point is important, since this implies that ADO does not induce biases in model selection.

## 4. Discussion

In this paper, we demonstrate the added-value of real-time acquisition and BCI loops, when applied to the aim of performing online design optimization. This work follows recent advances in Bayesian decision theoretic approaches to design optimization in experimental neuroscience [32]. We first validated our approach in the case of simple psychological models of memory retention [20,24,38]. We then extended our approach to more realistic and complex multi-model comparisons, given electrophysiological data. In brief, ADO outperforms classical (offline) designs, irrespective of the true generative process. This means we expect ADO to be most useful in experimental settings whose optimality cannot be known in advance, *i.e.*, when comparing complex models given low quality data (e.g., a low sample size and SNR (Signal-to-Noise Ratio)).

### 4.1. Current Limitations

First, ADO’s performances depend upon the accuracy of prior information regarding model parameters. In fact, non-informative priors are unacceptable, because they induce flat predictive densities for all models, which prevents any design optimization procedure [32]. A solution to this issue would be to start with a classical (offline) design, perform model inversions given the first few data samples and then use the ensuing posterior distributions as priors in ADO.

Second, real-time processing of electrophysiological data remains challenging, because of data contamination by high-magnitude artifacts (*i.e.*, muscle activity, head movements, eye blinks, *etc.*). This means one may have to deal with missing data. This may be problematic when dealing with dynamical models that assume some continuity in the processes underlying experimental data (e.g., belief update in learning experiments).

Third, ADO cannot be used to optimize the experimental design and to select relevant data features (e.g., EEG markers) at the same time. This implies that admissible data features have to be identified prior to the experiment.

### 4.2. Perspectives

A promising application of ADO is differential diagnosis, whereby one seeks to discriminate between alternative pathological mechanisms. One such example is the inference of patients’ mental states from electrophysiological makers in coma and related disorders [55,56,57]. Beyond such diagnostic objectives, ADO could prove useful in model-based predictions of individual treatment responses. Lastly, although BCI applications are often evaluated with respect to their clinical utility, we would like to emphasize that ADO (when combined with real-time electrophysiology) could find a wide range of practical applications in basic neuroscientific research. 

## 5. Conclusion

Our paper aims to provide a proof of concept of an original way to conduct basic research experiments. Using simulations, we demonstrated robust advantages of optimal design when the ADO procedure was compared with classical designs in behavioral or electrophysiological experiments. We envisage that the present paper could pave the way for future BCI applications in both basic and clinical research.

## Appendix A

### A1. Bayesian Inference

In this Appendix, we briefly describe how approximate Bayesian inference applies to dynamic causal models, with a particular emphasis on Bayesian model comparison.

In the Bayesian framework, defining model *M* amounts to defining the likelihood and prior densities, respectively *p*(*y│x, θ, φ, u, M*) and *p*(*x, θ, φ*│*M*). Solving Bayes rule then produces the posterior density, *p*(*x, θ, φ*│*y, M*), and the model evidence or marginal likelihood, *p*(*y*│*M*, *u*). The former enables inference on model parameters, while the later enables model comparison and selection. However, for complex non-linear models, such as DCMs, exact computation of those two quantities becomes intractable. Variational Bayes (VB) then offers a convenient and efficient approximate inference method, which operates iteratively and furnishes analytic forms to the posterior and model evidence. In short, it maximizes a lower bound to the model log-evidence, called the (negative) free energy, which writes:

*F* = log *p*(*y*│*u, M*) − *KL*(*q*(*x, θ, φ*)│*p*(*x, θ, φ*│*y, M*))
(11)

where *KL* is the Kullback–Leibler divergence between an approximate posterior, *q*, and the true posterior. Since *KL* is always positive, *F* is a lower bound to the model log-evidence and maximizing *F* amounts to minimizing *KL*, such that *q* gets closer to the true posterior, while *F* gets closer to the model log-evidence. At the convergence of the VB process, *F* is used for model comparison, and the approximate posterior, *q*, for the winning model is used for inference on the parameters. Importantly, it can be easily shown that the free energy criterion forms a trade-off between model accuracy and model complexity [59]. In other words, it implements the parsimony principle, or Occam’s razor, to prevent overfitting [60].

For a more detailed description of the VB approach and an exemplar application in neuroimaging, we refer the interested reader to [31] and [61], respectively.

In the Bayesian framework, comparing Model *M*_1_ with Model *M*_2_ rests upon computing the Bayes factor:

(12)

which simply corresponds to the ratio of the two model evidences. Like in classical inference, where decisions are made based on so-called *p*-values, similar decisions can be made based on Bayes factors [62]. Hence a BF (Bayes factor) greater than 20 (or a log-BF greater than three) is considered as strong evidence in favor of Model *M*_1_ (equivalently, to a *p*-value lower than 0.05 (=1/20)). When the dimension of the model space is larger than two, a convenient quantity is the model posterior, which can easily be derived from the Bayes rule as follows:

(13)

where:

(14)

Under equiprobable priors over models, this boils down, for Model, *M_k_* to:

(15)

Then, a natural decision criterion is to select as the best model the one that obtains a posterior probability greater than 0.95.

### A2. Design Efficiency: A Decision Theoretic Criterion

In this Appendix, we summarize the decision theoretic approach introduced in [32] to optimize design efficiency for the comparison of non-linear models of the sort described in Section 2.1.

In Section 2.1, we saw that model selection in a Bayesian framework involves evaluating model evidence. The reason why *p*(*y*│*m, u*) is a good proxy for the plausibility of any model, *M*, is that the data, *y*, sampled by the experiment is likely to lie within the subset of *Y* that is highly plausible under the model that makes predictions most identical to the true generative process of the data. However, there is a possibility that the experimental sample, *y*, would end up being more probable under a somewhat worse model. This “model selection error” could simply be due to chance, since *y* is sampled from a (hidden) probability distribution. The inferential procedure of Bayesian model selection should then be designed to minimize (in expectation) the above model selection error. The probability, *P_e_*, of selecting an erroneous model depends on both the data, *y*, that will be sampled and the experimental design, *u*, that will be used. It is given by:

(16)

The task of design optimization is to reduce the effect of the data sampling process upon the overall probability of selecting the wrong model. In other words, the design risk we want to minimize corresponds to the marginalization of the above probability over the whole sample space, *Y*. Our optimal design, *u*^*^, thus writes:

(17)

with:

(18)

Unfortunately, the above integral has no analytical close form and will be difficult to evaluate in most cases. As proposed in [32] instead, we minimize an information theoretic criterion, *b*(*u*), which yields both upper and lower bounds to the above error rate. *b*(*u*) is known as the Chernoff bound [63] and is such that:

(19)

with:

*b*(*u*) = *H*(*p*(*M*)) − *D_JS_* (*u*)
(20)

where *N_M_* is the cardinality of the model comparison set, *H*(∙) is the Shannon entropy and *D_JS_*(∙) is the Jensen–Shannon divergence [64], which is an entropic measure of dissimilarity between probability density functions (see Appendix A3). As shown in Appendix A3, this approximate criterion is very similar and brings a new perspective to the one initially proposed by [20].

### A3. Comparison of the Chernoff Bound with the Other Criterion

In this Appendix, we disclose the relationship between the Chernoff bound we use for online design optimization and the criterion proposed in the seminal work by Myung, Pitt and Cavagnaro [20].

The Chernoff bound writes (see Appendix A2):

*b*(*u*) = *H*(*p*(*M*)) − *D_JS_* (*u*)
(21)

Minimizing this bound to the model selection error rate, with respect to design variable *u*, is equivalent to maximizing the Jensen–Shannon divergence:

(22)

Simply unfolding Shannon’s entropy and applying Bayes rule yields:

(23)

where *I*(*M;Y*│*u*) is the mutual information between the data and model spaces, given experimental design *u*. This conditional mutual information is the information theoretic criterion derived and maximized by Myung and colleagues.

The Jensen-Shannon divergence, or equivalently, the above conditional mutual information, is the relevant terms of the Chernoff bound to the model selection error rate. For simple models, such as the memory retention models compared here and in [20], the model selection error rate can easily be computed with high precision using Monte-Carlo simulations. However, when comparing nonlinear models, such as the learning models considered in this paper, the appeal to the approximate design efficiency provided by the Jensen-Shannon divergence is required*.* Importantly for such models, under the Laplace approximation, this criterion takes a simple analytic form, which can be computed online efficiently [32].
